# Targeting Brain Disease in MPSII: Preclinical Evaluation of IDS-Loaded PLGA Nanoparticles

**DOI:** 10.3390/ijms20082014

**Published:** 2019-04-24

**Authors:** Laura Rigon, Marika Salvalaio, Francesca Pederzoli, Elisa Legnini, Jason Thomas Duskey, Francesca D’Avanzo, Concetta De Filippis, Barbara Ruozi, Oriano Marin, Maria Angela Vandelli, Ilaria Ottonelli, Maurizio Scarpa, Giovanni Tosi, Rosella Tomanin

**Affiliations:** 1 Department of Women’s and Children’s Health, University of Padova, 35128 Padova, Italy; laura.rigon@unipd.it (L.R.); elisalg@yahoo.it (E.L.); frale100@gmail.com (F.D.); concetta.defilippis93@gmail.com (C.D.F.); maurizio.scarpa@unipd.it (M.S.); 2 Fondazione Istituto di Ricerca Pediatrica “Città della Speranza”, 35127 Padova, Italy; marika.salvalaio@unipd.it (M.S.); francesca.pederzoli@unimore.it (F.P.); 3 Department of Pharmaceutical and Pharmacological Sciences, University of Padova, 35131 Padova, Italy; 4 Department of Life Sciences, University of Modena and Reggio Emilia, 41124 Modena, Italy; jasonthomas.duskey@unimore.it (J.T.D.); barbara.ruozi@unimore.it (B.R.); mariaangela.vandelli@unimore.it (M.A.V.); ilaria.ottonelli@unimore.it (I.O.); gtosi@unimore.it (G.T.); 5 Department of Biomedical Sciences, University of Padova, 35121 Padova, Italy; oriano.marin@unipd.it

**Keywords:** lysosomal storage disorders, mucopolysaccharidosis, Hunter syndrome, MPS II, enzyme replacement therapy, nanoparticles, PLGA, blood-brain barrier, central nervous system, brain targeting

## Abstract

Mucopolysaccharidosis type II (MPSII) is a lysosomal storage disorder due to the deficit of the enzyme iduronate 2-sulfatase (IDS), which leads to the accumulation of glycosaminoglycans in most organ-systems, including the brain, and resulting in neurological involvement in about two-thirds of the patients. The main treatment is represented by a weekly infusion of the functional enzyme, which cannot cross the blood-brain barrier and reach the central nervous system. In this study, a tailored nanomedicine approach based on brain-targeted polymeric nanoparticles (g7-NPs), loaded with the therapeutic enzyme, was exploited. Fibroblasts from MPSII patients were treated for 7 days with NPs loaded with the IDS enzyme; an induced IDS activity like the one detected in healthy cells was measured, together with a reduction of GAG content to non-pathological levels. An in vivo short-term study in MPSII mice was performed by weekly administration of g7-NPs-IDS. Biochemical, histological, and immunohistochemical evaluations of liver and brain were performed. The 6-weeks treatment produced a significant reduction of GAG deposits in liver and brain tissues, as well as a reduction of some neurological and inflammatory markers (i.e., LAMP2, CD68, GFAP), highlighting a general improvement of the brain pathology. The g7-NPs-IDS approach allowed a brain-targeted enzyme replacement therapy. Based on these positive results, the future aim will be to optimize NP formulation further to gain a higher efficacy of the proposed approach.

## 1. Introduction

Mucopolysaccharidosis type II (MPSII, MIM #309900) is a rare, inherited disorder belonging to the group of mucopolysaccharidoses, a subgroup of the lysosomal storage disorders (LSDs). MPSII is a devastating disease, due to the deficit of the lysosomal hydrolase iduronate 2-sulfatase (IDS, EC3.1.6.13), thus causing a pathological accumulation of the undegraded glycosaminoglycans (GAG) heparan- and dermatan-sulfate. Clinical phenotype involves dysfunction of most organs, including liver, spleen, heart, lungs, bones, joints, eyes, and ears [1,2]. In the severe forms, affecting about two-thirds of the patients, the brain is also severely compromised. Although the phenotype presents a continuum of forms, patients are commonly classified as severe or attenuated, mainly based on the presence/absence of a progressive neuro-degeneration [3].

Several therapeutic protocols have been tested for the disease in the last decades; among these, the most commonly applied is enzyme replacement therapy (ERT), consisting of a weekly infusion of the recombinant iduronate 2-sulfatase (IDS) [4]. Although ERT has shown some peripheral efficacy in patients [2,5,6,7], it cannot help the brain disease due to the inability of IDS, like other lysosomal enzymes, to cross the blood-brain barrier (BBB) and reach the brain tissue. Another approach, hematopoietic stem cell transplantation, has shown in MPSII an almost complete inefficacy on brain disease, and a quite high risk to benefits ratio; therefore, the procedure is scarcely applied [8].

In recent years, many studies have focused on the treatment of the neurological involvement in MPSII and on the development of brain-targeted therapies. In clinics, only the intrathecal delivery of IDS has been investigated [9], while several therapeutic approaches have been tested in the mouse model. These preclinical evaluations include: high systemic dosage of ERT [10], direct administration of IDS to the brain compartment by intracerebroventricular or intrathecal injections [11,12,13], gene therapy mediated by adeno-associated or lentiviral vectors encoding human IDS [14,15,16], substrate reduction therapy [17], and conjugation with brain-targeting ligands [18,19]. In many of these studies, various limitations related to safety or efficacy aspects have been highlighted, while some of them have shown to be more promising and deserving of a deeper analysis.

In general, due to the vulnerability of the brain compartment and the consequent need to maintain its integrity, among all possible strategies, non-invasive ones are to be preferred in developing therapies for the central nervous system (CNS). In the last decade, the nanomedicine-based approach was considered for neurological applications [20], as well as for LSDs treatment [21,22]. Different kinds of polymer can be used for nanoparticles preparation (e.g., poly-lactide-co-glycolide, polyethylene glycol, poly-buthyl/heaxil-cyano-acrylate, albumin, chitosan) as well as different types of inorganic materials (gold, silica, carbon) [23,24]. In the last years, several ligands have been used to modify nanoparticles and tested for specific receptor binding on the BBB (e.g., transferrin, low-density lipoprotein, nicotinic acetylcholine, integrin, insulin) [20]. Our group recently reported for the MPSI and MPSII mouse models a successful BBB crossing of polylactide-co-glycolide (PLGA) biocompatible and biodegradable nanoparticles, functionalized with a glycopeptide of 7 amino acids (g7-NPs) for CNS targeting, and loaded with a model drug, FITC-albumin [25]. We here describe the subsequent in vitro and in vivo experiments, conducted in MPSII cells and mouse model, to evaluate the efficacy of the brain-targeted NPs loaded with the recombinant IDS (g7-NPs-IDS).

## 2. Results and Discussion

### 2.1. NPs Characterization

All NPs prepared with the double emulsion technique were well formed, with a spherical shape, and were homogeneous as per the intra-batches analysis. The characterization in terms of size, surface charge, and drug loading (Table 1) showed that, independently from surface modification or loading, all NPs were featured by dimension around 200 nm, thus compatible with systemic administration, with good homogeneity and negative surface charges, as usual when considering PLGA-NPs [26]. The major difference was related to the different percentage in loading efficiency (EE%) of u-NPs loaded drug (30%) with respect to g7-NPs (15%), with a strong decrease in IDS content in g7-NPs-IDS with respect to u-NPs-IDS. As this data was confirmed in at least three different batches of NPs, we assumed that this difference could be due to amphiphilic properties of the g7 peptide linked to PLGA, which could act as a bridge between the oil and water phases and could compete with IDS site within the hydrophilic environment inside the polymeric matrix. This could affect the stability of the process of encapsulation, leading to loss of IDS in the outer water phase and to a higher variability in drug content. SEM and AFM images (Figure S1) demonstrated the homogeneity of both formulations in terms of shape and size.

### 2.2. In Vitro Analysis of u-NPs-IDS Efficacy

An in vitro study was performed to verify NPs capability to encapsulate, transport, and maintain therapeutic efficacy of the recombinant enzyme. The primary fibroblasts, collected from patients with MPSII and healthy subjects, were treated with free IDS, or IDS loaded in NPs (u-NPs-IDS) or left untreated. We chose to use only un-modified NPs as g7 peptide was already shown not to impact or decrease the NPs uptake in different cell models [27,28,29].

**Figure 1:**
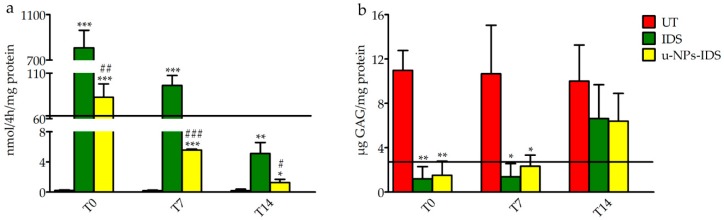
Induced IDS activity and GAG levels in treated fibroblasts. (**a**) Induced IDS activity expressed in nmoles of 4MU (4-Methylumbelliferyl) released in 4 h per mg of protein (nmol/4 h/mg protein) and (**b**) GAG content (μg GAG/mg protein) after 7 days treatment in fibroblasts from MPSII patients (*n* = 3). Type of treatment: untreated MPSII fibroblasts (UT), free IDS (IDS), untargeted NPs loaded with IDS enzyme (u-NPs-IDS). The solid horizontal line is the reference value of cells from healthy subjects (mean of 3 samples). The evaluation was carried out 0 (T0), 7 (T7), and 14 (T14) days after the end of the treatment. Data are mean ± SD. For each time point, asterisks indicate a statistically significant difference from UT cells (two-tailed Student’s *t*-test, * *p* < 0.05, ** *p* < 0.01, *** *p* < 0.001), while hash marks indicate a statistically significant difference between IDS and u-NPs-IDS treated cells (two-tailed Student’s *t*-test, ^#^ *p* < 0.05, ^##^ *p* < 0.01, ^###^ *p* < 0.001). Data are the result of two separate experiments in three different cell lines and each analysis has been repeated three times in duplicate.

After 7 days, the supplemented medium was removed and cells lysed at 0, 7, and 14 days post-treatment. The in vitro evaluation confirmed the ability of NPs to encapsulate IDS and to transport it into the fibroblasts, preserving its activity. Although cells treated with the u-NPs-IDS presented a lower induced enzyme activity compared to cells treated with free IDS (Figure 1a), such an induced activity reached the level normally measured in healthy control cells and it was sufficient to reduce the GAG content of the cells to non-pathological levels (Figure 1b).

### 2.3. In Vivo Analysis of g7-NPs-IDS Efficacy

An in vivo study was conducted in the MPSII mouse model, by treating mice once a week for 6 weeks. Following treatment, 5 mice/group (Ids-ko treated with 0.9% NaCl, g7-NPs, free IDS, g7-NPs-IDS and wt mice) were sacrificed and autoptic samples used for biochemical as well as histological and immunohistochemical evaluations.

#### 2.3.1. Histochemical and Biochemical Analysis of GAG Deposits

**Figure 2:**
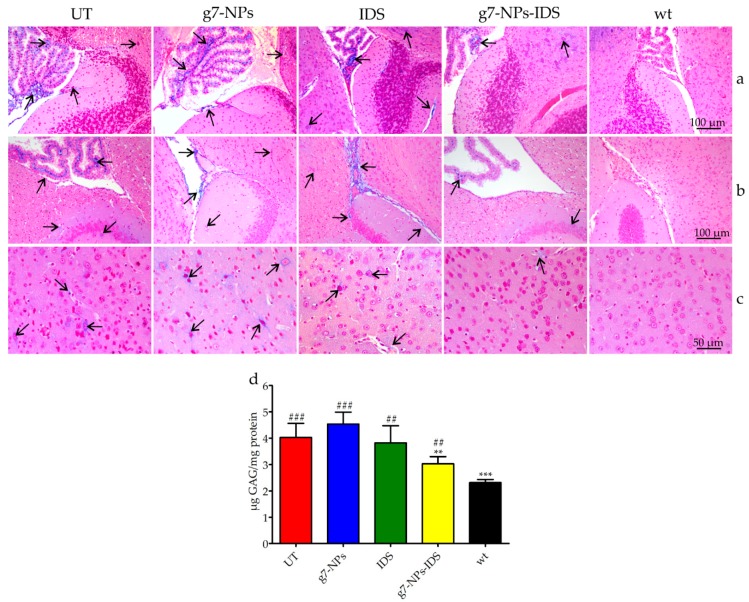
Analysis of GAG in the brain of treated Ids-ko mice. Histochemical and biochemical analysis of GAG in the brain of Ids-ko mice treated with 0.9% NaCl (untreated, UT), g7-NPs, free IDS, g7-NPs-IDS, and in wt mice, after 6 weeks of treatment. Representative sections of the brain storages in (**a**) cerebellum with the fourth ventricle, (**b**) hippocampus with the third ventricle, and (**c**) cerebral cortex. Arrows indicate GAG deposits. Sections (7 μm) were stained with 1% Alcian Blue pH 2.5, counterstained with 0.1% Nuclear Fast Red. (**d**) GAG content (μg GAG/mg protein) detected in the brain parenchyma after 6 weeks of treatment (*n* = 5 per treatment group). All values are represented as mean ± SD. Asterisks indicate a statistically significant difference from UT mice (Mann–Whitney *U* test, ** *p* < 0.01, *** *p* < 0.001); hash marks indicate a statistically significant difference from wt mice (Mann–Whitney U test, ^##^ *p* < 0.01, ^###^ *p* < 0.001).

**Figure 3:**
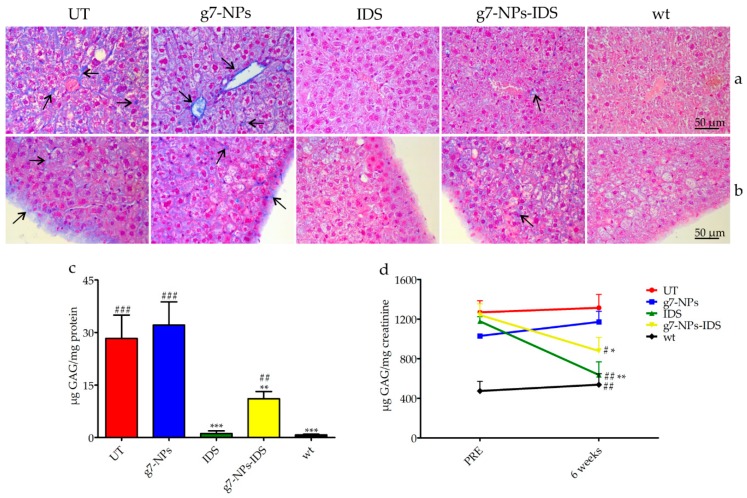
Analysis of GAG in the liver and urine of treated Ids-ko mice. Histochemical and biochemical analysis of GAG in liver and urine of Ids-ko mice treated with 0.9% NaCl (untreated, UT), g7-NPs, free IDS, g7-NPs-IDS, and in wt mice, after 6 weeks of treatment. Representative sections of (**a**) hepatic vessels and (**b**) Glisson’s capsule. Arrows indicate GAG deposits. Sections (7 μm) were stained with 1% Alcian Blue pH 2.5, counterstained with 0.1% Nuclear Fast Red. (**c**) GAG content (μg GAG/mg protein) detected in the liver after 6 weeks of treatment (*n* = 5 per treatment group). Data are mean ± SD. Asterisks indicate a statistically significant difference from UT mice (Mann–Whitney *U* test, * *p* < 0.05, ** *p* < 0.01, *** *p* < 0.001); hash marks indicate a statistically significant difference from wt mice (Mann–Whitney *U* test, ^##^ *p* < 0.01, ^###^ *p* < 0.001). (**d**) Urinary GAG content (µg GAG/mg creatinine) detected just before the start of treatment (PRE) and after 6 weeks of treatment (6 weeks) (*n* = 5 per treatment group). All values are represented as mean ± SD. Asterisks indicate a statistically significant difference between the same treatment-group before starting (PRE) and after 6 weeks of treatment (Mann–Whitney *U* test, *** *p* < 0.001); hash marks indicate a statistically significant difference of the samples obtained from mice after 6 weeks of treatment vs. age-matched UT mice (Mann–Whitney *U* test, ^#^ *p* < 0.05, ^##^ *p* < 0.01).

The results of the evaluation of GAG storage are shown in Figure 2 and Figure 3. Histochemical analysis of the brain GAG content is presented in Figure 2a–c. As previously reported [30], the major areas of GAG accumulation in the brain of UT mice were within the third and fourth ventricles, and at a lower extent in the cerebral cortex. The same deposits were visible also in g7-NPs and free IDS treated mice, while in the brain of g7-NPs-IDS treated mice a lower accumulation was observed and confirmed by the biochemical analysis of GAG content (Figure 2d). As previously reported by our group [17], in the liver of UT mice, GAG storage was highlighted in the mesothelial cells and in the Glisson’s capsule, and also around the vessel membrane of the portal tract and in the extracellular matrix of hepatocytes. A similar GAG distribution is visible both in the g7-NPs treated and in the free IDS treated mice; although still visible also in the liver of Ids-ko mice treated with g7-NPs-IDS, it is present at a visible lower extent (Figure 3a–c).

The biochemical analysis of GAG content confirmed data obtained by the histochemical evaluations. About 25% GAG decrease was detected in the brain parenchyma of Ids-ko mice treated with g7-NPs-IDS (Figure 2d), demonstrating the ability of these nanoparticles to transport the enzyme across the BBB, maintaining its therapeutic efficacy. As previously described for the mouse model of MPSIIIB [31], the animal model for MPSII also shows at baseline much lighter GAG deposits in the brain compared to other tissues, when a biochemical analysis is performed. Approximately, a two-fold increase in GAG level compared to wild-type mice was previously described [17,32] and confirmed (Figure 2d).

In the liver (Figure 3c), GAG deposits were on average 30 times higher in UT or g7-NPs treated Ids-ko vs. wt mice. A significant decrease of GAG content (about 60%) was obtained in mice treated with g7-NPs-IDS compared to UT mice, although this decrease was much lower than that observed in mice treated with free IDS (about 95%). This result could be partially due to the inability of the free IDS to cross the BBB, and therefore to its greater bioavailability in the other organs, including the liver, resulting in greater activity and efficacy in this district.

Figure 3d shows the urinary GAG content before and after 6 weeks of treatment. With respect to the starting point (PRE) and to UT mice, mice treated with either free IDS or g7-NPs-IDS showed a statistically significant decrease in GAG content.

The results of both biochemical and histochemical GAG evaluation in brain parenchyma, liver, and urine showed the ability of g7-NPs-IDS to significantly reduce these pathological storages, although the reduction obtained was not enough to normalize GAG to healthy animal levels. The NPs treatment turned out to be less efficacious compared to free IDS, except for brain tissue, unreachable by the free enzyme.

#### 2.3.2. Histological and Immunohistochemical Brain Analysis

**Figure 4:**
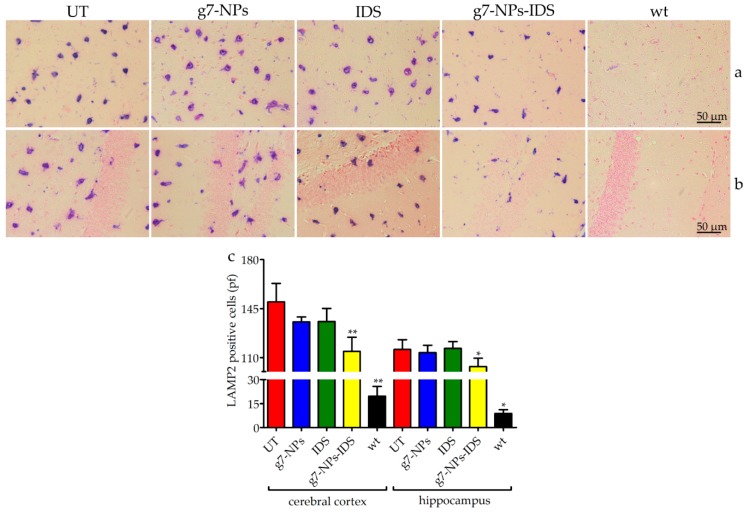
Immunohistochemical analysis of LAMP2 in the brain. Immunohistochemical analysis of LAMP2 in Ids-ko mice treated with 0.9% NaCl (untreated, UT), g7-NPs, free IDS, g7-NPs-IDS, and in wt mice, after 6 weeks of treatment. Representative images of (**a**) cerebral cortex and (**b**) hippocampus of 7 µm sections stained with LAMP2 antibody (purple spots) and counterstained with 0.5% Eosin Y solution. (**c**) Quantification of positive cells to LAMP2 in the cerebral cortex and hippocampus. *n* = 5 mice/group, pf = per field. All data are mean ± SD. Asterisks indicate a statistically significant difference from UT mice (Mann–Whitney *U* test, * *p* < 0.05, ** *p* < 0.01). All Ids-ko mice groups presented a statistically significant difference from wt mice.

The increase/reduction of glycosaminoglycan storage within lysosomes can be indirectly assessed through the analysis of lysosomal membrane proteins; therefore sections of paraffin embedded-brains were stained by using the lysosomal associated membrane protein 2 (LAMP2) antibody (Figure 4a,b). As shown in the panel, positive lysosomal signals were detected both in the cerebral cortex (Figure 4a) and in the hippocampus (Figure 4b) of all Ids-ko mice. The number of cells positive to LAMP2 (Figure 4c) showed a significant tendency to decrease in the cerebral cortex and in the hippocampus of the animals treated with g7-NPs-IDS (respectively 24% and 10% vs. UT mice, with *p* < 0.01 and *p* < 0.05), consistently with the data obtained from the biochemical and histological/immunohistochemical analyses of GAG. As expected, no significant decreases were observed in mice treated with g7-NPs and free IDS.

Figure S2 shows representative sections of the cerebellum stained with 0.1% Toluidine Blue O solution. Vacuolation was detected in Purkinje cells of UT, g7-NPs and IDS-treated mice, whereas treatment with g7-NPs-IDS showed a slight reduction of vacuolation, indicating that the enzyme delivered to the brain was able to partly correct the damage at this level, perhaps suggesting that the complete correction may require a longer treatment and preferably earlier in life.

#### 2.3.3. Evaluation of the Neuroinflammation

**Figure 5:**
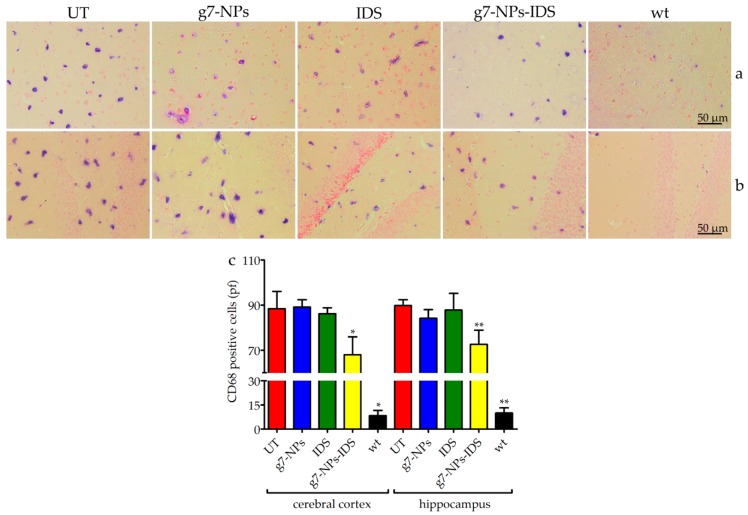
Immunohistochemical analysis of CD68 in the brain. Immunohistochemical analysis of CD68 in Ids-ko mice treated with 0.9% NaCl (untreated, UT), g7-NPs, free IDS, g7-NPs-IDS, and in wt mice, after 6 weeks of treatment. Representative images of (**a**) cerebral cortex and (**b**) hippocampus of 7 µm sections stained with CD68 antibody (purple spots) and counterstained with 0.5% Eosin Y solution. (**c**) Quantification of positive cells to CD68 in the cerebral cortex and hippocampus. *n* = 5 mice/group, pf = per field. All data are mean ± SD. Asterisks indicate a statistically significant difference from UT mice (Mann–Whitney *U* test, * *p* < 0.05, ** *p* < 0.01). All Ids-ko mice groups presented a statistically significant difference from wt mice.

**Figure 6:**
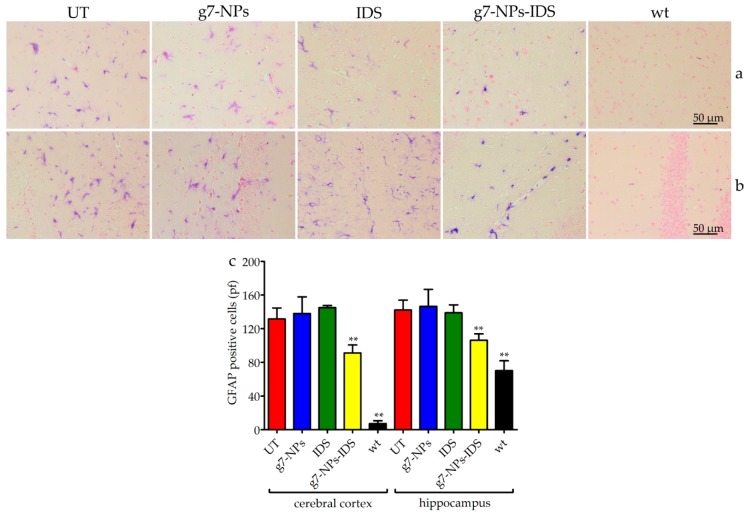
Immunohistochemical analysis of GFAP in the brain. Immunohistochemical analysis of GFAP in Ids-ko mice treated with 0.9% NaCl (untreated, UT), g7-NPs, free IDS, g7-NPs-IDS and in wt mice, after 6 weeks of treatment. Representative images of (**a**) cerebral cortex and (**b**) hippocampus of 7 µm sections stained with GFAP antibody (purple spots) and counterstained with 0.5% Eosin Y solution. (**c**) Quantification of positive cells to GFAP in the cerebral cortex and hippocampus. *n* = 5 mice/group, pf = per field. All data are mean ± SD. Asterisks indicate a statistically significant difference from UT mice (Mann–Whitney *U* test, ** *p* < 0.01). All Ids-ko mice groups presented a statistically significant difference from wt mice.

To determine whether nanoparticles could reduce neuroinflammation in Ids-ko mice, we evaluated the number of positive microglial cells for CD68 (a marker for microgliosis) (Figure 5) and the number of positive astrocytes for GFAP (a marker for astrogliosis) (Figure 6) in the cerebral cortex and hippocampus sections of treated mice. UT, g7-NPs, and IDS treated mice exhibited a marked increase in neuroinflammatory microglial cells and astrocytes compared to wt (about 11-fold for microglial cells in both areas, and about 16-fold for astrogliosis in the cerebral cortex and 2-fold in the hippocampus). Instead, g7-NPs-IDS treatment significantly reduced neuroinflammation in microglia and astrocytes in both analyzed areas (20% vs. UT mice for CD68, with *p* < 0.05 in the cerebral cortex and *p* < 0.01 in the hippocampus; 25% vs. UT mice for GFAP with *p* < 0.01 in both areas).

The reduction in the number of CD68 and GFAP positive cells fully reflects the trend of GAG storage in the brain parenchyma, confirming the ability of the g7-NPs to transport the IDS enzyme beyond the BBB, otherwise uncrossable. This last data is here confirmed by the comparable negative results obtained in the Ids-ko animals treated with either free IDS enzyme or empty g7-NPs.

Overall, results obtained with this short-term study are encouraging since they show for the first time an efficacious brain targeting of the IDS enzyme by using a non-invasive delivery system. Although the obtained improvement of the brain disease could not reach full normalization, the positive results achieved strongly encourage pursuing this strategy by optimizing NPs formulation, possibly increasing their enzyme uploading and stability, allowing a higher efficacy of this therapeutic approach in the brain district.

## 3. Materials and Methods

### 3.1. Chemicals

Poly(D,L-lactide-co-glycolide) (PLGA, RG503H, MW near 11,000) was used as received from the manufacturer (Boehringer-Ingelheim, Ingelheim am Rhein, Germany). Polyvinyl alcohol (PVA, MW 15,000) was purchased from Sigma-Aldrich (Milan, Italy). Gly-L-Phe-D-Thr-Gly-L-Phe-L-Leu-L-Ser(O-β-D-Glucose)-CONH2 (g7) linked to PLGA was synthesized as previously reported [26] and purchased from Mimotopes (Clayton, Victoria, Australia). Trehalose dihydrate (MW 378.33) was purchased from Sigma-Aldrich and used as cryoprotectant. A MilliQ water system (Millipore, Bedford, MA, USA) supplied with distilled water provided high-purity water (18 MΩ). All the other chemicals were of analytical grade.

### 3.2. Nanoparticles Preparation and Chemico-Physical Characterization

To prepare IDS-loaded NPs, double emulsion technology was exploited: 500 μL of Elaprase® (Shire, Lexington, MA, USA) in deionized water (final concentration 10 mg/mL) was emulsified to 2.5 mL CH_2_Cl_2_ solution of polymer (50 mg, 90% PLGA + 10% g7-PLGA) under cooling (5 °C) by using a probe sonicator (Microson Ultrasonic cell disruptor, Misonix Inc. Farmingdale, NY, USA) at 80 W for 45 s. The first inner emulsion was rapidly added to 8 mL of 1% (*w*/*v*) PVA aqueous solution and the w/o/w emulsion formed under sonication (80 W for 45 s) at 5 °C.

Formulation was mechanically stirred (1500 rpm) for at least 1 h (RW20DZM, Janke & Kunkel, IKA-Labortechnik, Staufen, Germany) at RT until the complete solvent evaporation, and finally purified by Hi-Speed Refrigerated Centrifugation (Beckman J21) at 17,000 rpm for 10 min at 5 °C, washed several times with water and re-suspended in water. With this technology, g7-NPs-IDS were obtained. The same procedure was applied to the preparation of unmodified NPs (u-NPs-IDS) used in the in vitro study by using 100% of PLGA as polymer, and also to the preparation of unloaded NPs (g7-NPs) used as control in the in vivo study and obtained using 500 µL of buffer solution instead of enzyme’s solution in the first emulsion. All the NP formulations, frozen using trehalose as a cryoprotectant (1:1 *w*/*w* polymer/trehalose ratio) and stored at −20 °C, were then characterized for surface, chemico-physical, and morphological properties as reported in the Supplementary Materials.

### 3.3. IDS Content

Freeze-dried NPs (5 mg) were dissolved in 1 ml of DCM. Then, 3 mL of PBS pH 7.4 were added to extract the IDS and the organic solvent was evaporated at RT under stirring (1500 rpm for at least 1 h; RW20DZM, Janke&Kunkel, IKA-Labortechnik). The aqueous solution was filtered (cellulose acetate filter, porosity 0.2 μm, Sartorius) to remove the polymer residues and spectrophotometrically analyzed at 492 nm to evaluate IDS concentration. The drug loading was expressed as mg of IDS encapsulated/100 mg of NPs and encapsulation efficiency (EE%), i.e. the percentage of encapsulated drug related to the initial amount of drug used in the preparation.

### 3.4. Cell Culture

Human fibroblasts from skin biopsies of 3 MPSII patients were obtained from the Telethon Biobank (Gaslini Institute, Genova, Italy). As controls, fibroblasts obtained from the circumcision of three healthy children were used. Written informed consent was acquired from patients at the time of biopsy. All cells were anonymously provided. They were expanded and maintained in cell culture under standard conditions: 37 °C in 5% CO_2_, in RPMI enriched with 15% fetal bovine serum (FBS), 100 U/ml penicillin, and 100 ng/mL streptomycin, L-Glutamine (all reagents from Thermo Fisher Scientific, Monza, Italy).

Cells were treated either with 15 nM free IDS or with 15 nM IDS encapsulated in NPs (u-NPs-IDS) or were left untreated. After 7 days, treatment was removed, and cells harvested immediately (T0) or after 7 or 14 additional days (T7 and T14). At any time-point, cell pellets were washed with 0.9% NaCl and then re-suspended in the same solution, sonicated, and evaluated for induced IDS activity and GAG content.

### 3.5. Mouse Model

The C57BL/6 Ids knockout (Ids-ko) mouse providing the model for MPSII was a kind gift from Joseph Muenzer (University of North Carolina, NC, USA) and it was generated by gene disruption of the murine Ids gene and previously characterized [30,32,33]. Mice were expanded in our animal facility and housed in light- and temperature-controlled conditions, with food and water provided ad libitum. This study was carried out in strict accordance with the European Directive 2010/63/EU. The protocol was approved by the Ethics Committee for Animal Experimentation of the University of Padova and authorized by the Italian Ministry of Health (CEASA project n.2/2013, approved on 25 March 2013).

In this study, experiments were performed in hemizygous affected and wild-type (wt) male mice, 12 weeks old at the beginning of the study, and an average weight of 28 g. Five Ids-ko mice were treated with g7-NPs-IDS (corresponding to 0.5 mg/kg/week of IDS, the same dosage administered to MPSII patients, and 32 mg/kg/week of NPs) once a week for 6 weeks, by intravenous injections. As controls, 5 Ids-ko mice injected with 0.9% NaCl (untreated, UT), 5 Ids-ko mice treated with unloaded g7-NPs (32 mg/kg/week), 5 Ids-ko mice treated with free IDS (0.5 mg/kg/week), and 5 wt untreated mice were also analyzed (Table S1). All mice were injected into the lateral caudal vein with an average volume of 120 µL for each treatment. Before starting treatment (PRE) and 6 weeks post-treatment, urine samples were collected using metabolic cages for 24 h and analyzed for GAG content. Mice were sacrificed by cervical dislocation after 6 weeks of treatment. Livers were collected and analyzed for GAG content. Half brain parenchyma was obtained after depletion of the capillary fraction and analyzed for GAG content as well. For each mouse, half brain and a liver lobe were collected and fixed in Bouin’s solution, for subsequent histochemical analyses.

### 3.6. Brain-Capillary Depletion

Brains were separated in the parenchyma and capillary fractions, using a dextran density centrifugation gradient, as previously described by Triguero [34]. The brain was homogenized at 4 °C in a physiological buffer at pH 7.4, containing: 10 mM HEPES, 141 mM NaCl, 4 nM KCl, 2.8 mM CaCl_2_, 1 mM MgSO_4_, 1 mM NaH_2_PO_4_, and 10 mM D-glucose. Dextran solution (AppliChem GmbH, Darmstadt, Germany) was then added to a final concentration of 19% and further homogenized. Parenchyma and capillary fractions were finally separated by centrifugation at 5000 *g* for 15 minutes at 4 °C.

### 3.7. IDS Enzyme Assay

Cell pellets were sonicated in 0.9% NaCl; after centrifugation, supernatants were recovered and protein concentration determined using the Bio-Rad Protein Assay (Bio-Rad Laboratories, Milan, Italy). IDS activity was evaluated by performing a fluorometric assay [35] employing the substrate 4-Methylumbelliferyl a-L-idopyranosiduronic acid 2-sulphate disodium salt (Moscerdam Substrates, Erasmus University, Rotterdam, The Netherlands), as previously described [36].

### 3.8. Measurement of Cells, Tissues, and Urinary GAG Content

Cell pellets were treated as described above for enzymatic analysis. Tissues were lyophilized, homogenized in 0.9% NaCl + 0.2% Triton X-100 (PanReac AppliChem GmbH) by a Polytron® PT1200E Disperser (Kinematica AG, Luzern, Switzerland), then left under stirring overnight at 4 °C, centrifuged at 1000 *g* for 5 min and the supernatant was recovered. Protein concentration was determined using the Bio-Rad Protein Assay. GAG content was measured by using Bjornsson’s protocol [37] with modifications, as previously described [17]. Urinary GAG content was determined using the protocol described by de Jong and colleagues [38] with modifications, as previously described [17].

### 3.9. Alcian Blue and Toluidine Staining and Immunohistochemistry

Upon mice sacrifice, tissues were dissected and fixed for 48 h in Bouin’s solution. They were then washed in 70% ethyl alcohol, dehydrated through a 70–100% ethanol gradient, clarified by xylene and paraffin embedded. 7 µm sections were then deparaffinized and stained with: 1% Alcian Blue pH 2.5/0.1% Nuclear Fast Red or 0.1% Toluidine Blue O pH 4.2 (all dyes from Sigma-Aldrich).

For immunohistochemistry, 7 µm thick serial sections were treated for antigen retriever with a 10mM sodium citrate buffer pH 6.0, for 15 min at 100 °C in a moist environment and then incubated for 1 h with blocking buffer (10% FBS in PBS). Overnight incubation with primary antibodies was performed using rat anti-LAMP2 (1:250, ab13524, Abcam, Cambridge, UK), rabbit anti-GFAP (1:250, OPA1-06100, Thermo Fisher Scientific) and rabbit anti-CD68 (1:250, PA1518-1, BosterBio, Pleasanton, CA, USA). After washing with TBS-T, secondary alkaline-phosphatase-conjugated antibodies were incubated for 1 h at room temperature (1:1000 donkey anti-rat, A18748, Thermo Fisher Scientific; 1:1000 donkey anti-rabbit, ab97082, Abcam). The color was developed for about 15 minutes using the NBT/BCIP (nitro-blue tetrazolium chloride/5-bromo-4-chloro-3′-indolyphosphate p-toluidine salt) method (SERVA Electrophoresis GmbH, Heidelberg, Germany). Finally, a counterstain with 0.5% Eosin Y solution was performed and sections were mounted with Eukitt® (both Sigma-Aldrich).

### 3.10. Microscopy Analysis

Visible analysis was performed using a Leica DM LB/30 microscope. All samples were blindly analyzed and quantified using ImageJ software (http://imagej.nih.gov/ij) on at least 3 images per area per 4 slices (total counted images = 12) for each mouse. The analyses were carried out on acquisitions at 20× magnification, while the panels show images with a 40× magnification for a better representation.

### 3.11. Statistical Analysis

Statistically significant differences between groups were determined by applying two-tailed Student’s *t*-test for the in vitro analysis and the non-parametric Mann–Whitney *U* test for the in vivo analysis, using GraphPad Prism 5 software (La Jolla, CA, USA). Significance was set at *p* < 0.05. Data are presented as mean ± standard deviation. All biochemical analyses were repeated at least 3 times in duplicate.

## 4. Conclusions

The delivery of active molecules across the BBB is now one of the most challenging issues in neuroscience research because it is estimated that more than 98% of the drugs are not able to reach an efficacious concentration in the brain compartment due to their chemico-physical characteristics [39]. To get access to innovative strategies for brain targeting, non-invasive techniques should be primarily investigated; among them, the nanomedicine-based approaches surely represent one of the most promising.

In this study, we evaluated the ability of brain-targeted nanoparticles (g7-NPs), already tested by our group with a high molecular weight model drug in MPSII [25], to bypass the BBB and deliver the IDS enzyme to the CNS, after systemic administration in the mouse model. A slight, but significant reduction of GAG storage in brain, liver, and urine was here demonstrated for the first time in mice treated with g7-NPs-IDS.

In addition to the accumulation of mucopolysaccharides, neuroinflammation has also been previously reported as a relevant aspect of the neuropathology of the MPSII mouse model, evaluated both by immunohistochemistry and by RNA-seq analyses [40,41]. In the present work, we demonstrated that g7-NPs-IDS could also ameliorate this aspect. In fact, we here showed a significant reduction in the number of positive microglial cells for CD68 and positive astrocytes for GFAP, hopefully indicating a general improvement of the neurological involvement following CNS-targeting of the IDS enzyme.

Based on the present results, we can conclude that a major step towards the treatment of the neurological aspect of MPSII has been made, though further efforts are still needed to optimize the strategy, aiming at future clinical applicability. Therefore, the next step will be to improve further the design of NPs formulations for a more efficient and efficacious IDS targeting of the brain compartment. Following optimization, it will be certainly of primary importance to carry out a long-term study to evaluate their long-term effects on disease development and progression, both from a neurological and a systemic point of view. In addition, given that other districts are currently scarcely treatable with ERT, as heart and bone, a possible future targeting of nanoparticles to these organs would represent an important challenge.

## Figures and Tables

**Table 1:** Physico-chemical characterization of PLGA nanoparticles. PDI = poly dispersity index; ζ-pot = zeta potential indicating surface charge; EE = encapsulation efficiency. a = values are given as mean ± SD (*n* = 9); b = the percentage of encapsulation efficiency was determined as the ratio of the encapsulated out of the total (encapsulated + free) drug percent (%). u-NPs-IDS = untargeted nanoparticles loaded with IDS enzyme; g7-NPs-IDS = brain-targeted nanoparticles loaded with IDS enzyme; g7-NPs= unloaded targeted NPs. Values are intended as mean ± SD (*n* = 9).

Samples	Z-Average ^a^ nm	PDI ^a^	ζ-pot ^a^ mV	mg of IDS/100 mg NPs	EE% ^b^
u-NPs-IDS	205 (12)	0.190 (0.02)	−36 (3)	3.1 (0.3)	31%
g7-NPs-IDS	203 (11)	0.214 (0.03)	−34 (5)	1.5 (0.9)	15%
g7-NPs	197 (12)	0.182 (0.01)	−32 (4)	/	/

## Supplementary Materials

Supplementary materials can be found at https://www.mdpi.com/1422-0067/20/8/2014/s1.

## Abbreviations

MPS	Mucopolysaccharidosis
MPSII	Mucopolysaccharidosis type II
IDS	Iduronate 2-sulfatase
NPs	nanoparticles
g7	Gly-L-Phe-D-Thr-Gly-L-Phe-L-Leu-L-Ser(O-β-D-Glucose)-CONH2
g7-NPs	Brain targeted NPs
g7-NPs-IDS	g7-NPs loaded with IDS enzyme
LSDs	Lysosomal storage disorders
GAG	Glycosaminoglycan
ERT	Enzyme replacement therapy
BBB	Blood-brain barrier
CNS	Central nervous system
PLGA	Poly(D,L-lactide-co-glycolide)
Ids-ko	Ids knockout
Wt	Wild-type
UT	Untreated
PVA	Polyvinyl alcohol
PBS	Phosphate buffer saline
FBS	Fetal bovine serum
NBT/BCIP	nitro-blue tetrazolium chloride/5-bromo-4-chloro-3′-indolyphosphate p-toluidine salt
