# Supplementary material for: Targeting Brain Disease in MPSII: Preclinical Evaluation of IDS-Loaded PLGA Nanoparticles
Source: Int J Mol Sci. 2019 Apr 24;20(8):2014. doi: 10.3390/ijms20082014 (PMC6514713; doi:10.3390/ijms20082014)
Supplement: Supplementary file 1 [file ijms-20-02014-s001.pdf]

International Journal of
Molecular Sciences

MDPI

SUPPLEMENTRAY METHODS

Chemico-physical characterization of NPs

For surface properties (size and charge), all the different batches of NPs suspended in distilled water were analyzed by photon correlation spectroscopy (PCS) and laser Doppler anemometry using a Zetasizer Nano ZS (Malvern, UK; Laser 4 mW He–Ne, 633 nm, Laser attenuator Automatic, transmission 100% to 0.0003%, Detector Avalanche photodiode, Q.E. > 50% at 633 nm, T = 25°C). Results were normalized with respect to a polystyrene standard solution.

The morphology and the structure/architecture of NPs were analyzed by scanning transmission electron microscopy (STEM). Briefly, a drop of a suspension of the samples was placed on a 200-mesh copper grid (TABB Laboratories Equipment, Berks, UK), allowed to adsorb, and the suspension surplus was removed by filter paper. All grids were analyzed using a Nova NanoSEM 450 (FEI, Oregon, USA) transmission electron microscope operating at 30 kV using a TEM grid holder and a STEM II detectors in Bright fields (BF) mode to analyze transmitted electrons.

Moreover, Atomic Force Microscopy (AFM) was used for morphological assessments. AFM was used (Park Instruments, Sunnyvale, CA, USA) at RT (about 20°C), at atmospheric pressure (760 mmHg) operating in air and in non-contact mode, using a commercial silicon tip-cantilever (tip diameter ≈5-10 nm) with stiffness about 40 Nm-1 and a resonance frequency around 150 kHz. A little amount of each NP sample was dispersed in deionised water (about 40 µL) on a small freshly cleaved mica disk (1 cm x 1 cm). Two min after deposition, the excess of deionised water was removed by a blotting paper and the sample observed. The topographical AFM images were obtained with a scan rate of 1 Hz and processed using ProScan Data Acquisition software developed under Windows 95.

As reported in a number of previous reports (Tosi et al 2007; Salvalaio et al 2016), the presence of g7 on NPs surface was confirmed by electron spectroscopy for chemical analysis (ESCA), showing the presence of nitrogen atoms due to g7 peptide onto the g7-NPs surface (data not shown). ESCA was performed on an XRC 1000 X-ray source analysis system (Specs Surface Nano Analysis, Germany) and a Phoibos 150 hemispherical electron analyzer (Specs Surface Nano Analysis, Germany), using MgKα1,2 radiations. Spectra were recorded in fixed retardation ratio (FAT) mode with 40 eV pass energy. The pressure in the sample analysis chamber was around 10−9 mbar. Data were acquired and processed using the SpecsLab2 software.

SUPPLEMENTARY DATA

Group name	Type and n. of mice	IDS injected	NPs injected
UT (pathological control)	5 Ids-ko	/	/
g7-NPs	5 Ids-ko	/	32 mg/kg/week (corresponding to 0.9 mg NPs/mouse/week)
IDS	5 Ids-ko	0.5 mg/kg/week (corresponding to 14 µg IDS/mouse/week)	/
g7-NPs-IDS	5 Ids-ko	0.5 mg/kg/week (corresponding to 14 µg IDS/mouse/week)	32 mg/kg/week (corresponding to 0.9 mg NPs/mouse/week)
wt (healthy control)	5 wt	/	/

Table S1: Mice injected in the *in vivo* study. UT = Ids-ko mice treated with 0.9% NaCl as pathological control; g7-NPs = Ids-ko mice treated with 32 mg/kg/week of g7-NPs; free IDS = Ids-ko mice treated with 0.5 mg/kg/week of IDS; g7-NPs-IDS = Ids-ko mice treated with 0.5 mg/kg/week of IDS and 32 mg/kg/week of g7-NPs; wt = wild-type mice used as healthy controls.

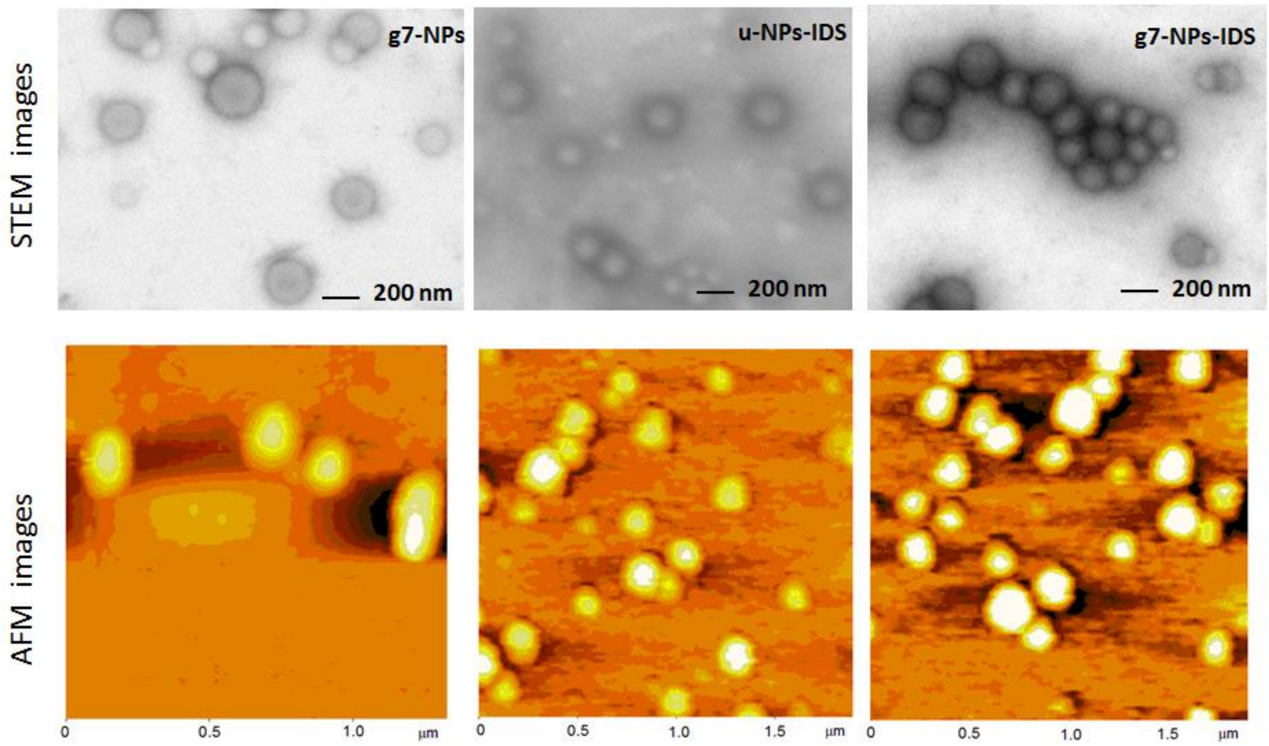

Figure S1: STEM and AFM analyses of g7-NPs, u-NPs-IDS, g7-NPs-IDS

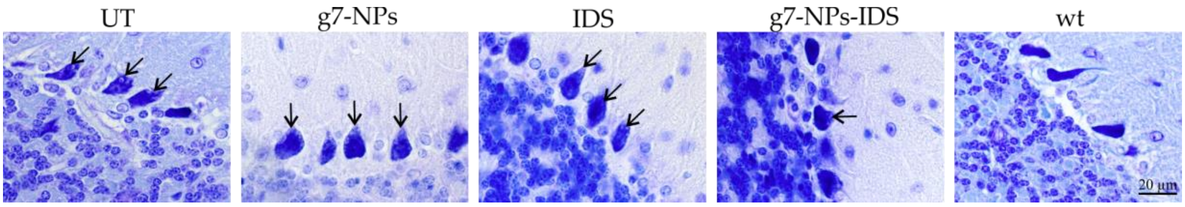

Figure S2: Toluidine staining of the cerebellum. Histochemical analysis of the cerebellum of Ids-ko mice treated with 0.9% NaCl (untreated, UT), g7-NPs, free IDS, g7-NPs-IDS and in the wt mice after 6 weeks treatment. Representative sections stained with 0.1% toluidine solution. Arrows indicate vacuolated Purkinje cells.
